# Prevalence, Risk Factors, and Comorbidities of Obstructive Sleep Apnea Risk Among a Working Population in Kuwait: A Cross-Sectional Study

**DOI:** 10.3389/fneur.2021.620799

**Published:** 2021-04-06

**Authors:** Husain Al-Qattan, Hamad Al-Omairah, Khaled Al-Hashash, Fahad Al-Mutairi, Mohammad Al-Mutairat, Mohammad Al-Ajmi, Anwar Mohammad, Abdulmohsen Alterki, Ali H. Ziyab

**Affiliations:** ^1^Department of Nuclear Medicine, Adan Hospital, Ministry of Health, Kuwait City, Kuwait; ^2^Department of Ophthalmology, Al-Bahar Eye Center, Ministry of Health, Kuwait City, Kuwait; ^3^Department of Obstructive and Gynecology, Maternity Hospital, Ministry of Health, Kuwait City, Kuwait; ^4^Department of Internal Medicine, Farwaniya Hospital, Ministry of Health, Kuwait City, Kuwait; ^5^Al-Rawda Health Center, Ministry of Health, Kuwait City, Kuwait; ^6^Al-Shuhada Health Center, Ministry of Health, Kuwait City, Kuwait; ^7^Department of Biochemistry and Molecular Biology, Dasman Diabetes Institute, Kuwait City, Kuwait; ^8^Department of Otolaryngology, Head, and Neck Surgery, Zain and Al-Sabah Hospitals, Ministry of Health, Kuwait City, Kuwait; ^9^Medical Division, Dasman Diabetes Institute, Kuwait City, Kuwait; ^10^Department of Community Medicine and Behavioral Sciences, Faculty of Medicine, Kuwait University, Kuwait City, Kuwait

**Keywords:** obstructive sleep apnea, risk factors, comorbidities, prevalence, Kuwait, adults

## Abstract

**Background:** Obstructive sleep apnea (OSA) affects a considerable proportion of adults globally and is associated with elevated morbidity and mortality. Given the lack of epidemiologic data on the burden of OSA in Kuwait, this study sought to estimate its prevalence, associated risk factors, and comorbid conditions among a working population in Kuwait.

**Methods:** This was a cross-sectional study of a sample of working adults (*n* = 651) from public institutions in Kuwait. High/low risk for OSA was ascertained according to the Berlin Questionnaire criteria. Participants self-reported their coexisting health conditions. Associations were assessed using Poisson regression with robust variance estimation; adjusted prevalence ratios (aPRs) and 95% confidence intervals (CIs) were estimated.

**Results:** Overall, 20.0% (130/651) of participants were classified as being at high risk for OSA, with more male than female subjects being at high risk (24.0% [56/233] vs. 17.7% [74/418], *P* = 0.053), though this difference did not gain statistical significance. Moreover, a high risk for OSA was more common among older and obese subjects. Factors associated with increased prevalence of a high risk for OSA included current smoking status (aPR = 1.58, 95% CI: 1.02–2.06), longer hours spent watching television (1.76, 1.10–2.81), and lower self-perceived physical health (2.11, 1.15–3.87). However, decreasing trends in the prevalence of high risk for OSA were observed with frequent engagement in vigorous physical activity and longer nightly sleep duration. Compared to those at a low risk for OSA, the subjects at high risk for OSA were more likely to have insomnia disorder (2.83, 1.81–4.41), diabetes (1.94, 1.15–3.27), hypertension (3.00, 1.75–5.16), and depression (4.47, 1.80–11.08).

**Conclusion:** This study estimated that 1/5 of working adults in Kuwait were at high risk for OSA, and the prevalence varied according to personal characteristics and lifestyle factors. Also, a high risk for OSA classification was associated with multiple comorbid health conditions.

## Introduction

Sleep disorders are broadly defined as medical disorders affecting sleep quality and quantity that are associated with increased morbidity and mortality, reduced quality-of-life, and impaired physical and mental functioning (1, 2). Signs and symptoms of sleep disorders may include excessive daytime sleepiness, irregular breathing or increased movement during sleep, and difficulty falling asleep and maintaining sleep (1, 3). Obstructive sleep apnea (OSA) is a common and major sleep disorder that is characterized by frequent and repeated episodes of partial (hypopnea) or complete (apnea) collapses of the upper airway during sleep that interrupt normal breathing (4). The most common cause that leads to arousal from sleep and/or oxygen desaturation during OSA events is oropharyngeal collapse at the back of the throat (4, 5), which results from a combination of anatomical factors that predispose the upper airway to collapse during inspiration and an insufficiency of neuromuscular compensation to maintain airway patency during sleep (6, 7). Snoring is the most common symptom experienced during sleep that is associated with OSA, whereas daytime symptoms include fatigue, excessive sleepiness, morning headaches, and poor concentration (5, 7, 8). Collectively, if left untreated, OSA can have serious and life-shortening consequences.

Worldwide, OSA remains a major source of morbidity and mortality, although estimates of its prevalence vary depending on personal characteristics (e.g., age, sex, obesity, race/ethnicity), as well as the methods and definitions used to ascertain OSA status (9, 10). Based on polysomnography assessment, prevalence estimates of mild OSA (apnea-hypopnea index [AHI] ≥5 to <15) have been shown to range from 9 to 38%, and estimates of the prevalence of moderate-to-severe OSA (AHI ≥15) vary from 6 to 17% among adults in the general population (9, 11). In large epidemiologic studies, the use of polysomnography is constrained by cost and logistics; hence, several questionnaires have been developed to identify subjects at a “high risk for OSA” in large population-based studies. These include the Berlin Questionnaire, the STOP questionnaire, the STOP-Bang questionnaire, and the Epworth Sleepiness Scale, with each instrument demonstrating differing accuracy (12–14).

Several unmodifiable and modifiable risk factors have been identified that influence the development of OSA. Unmodifiable risk factors include older age, male sex, race/ethnicity, family history of OSA, menopause in women, and craniofacial abnormalities (5, 15–17). The major modifiable risk factors of OSA include obesity, smoking, and alcohol use (5, 17, 18). Moreover, OSA has been linked with several comorbid conditions, such as stroke, myocardial infarction, hypertension, hyperlipidemia, diabetes, depression, and cognitive impairment (5, 6, 18–21). Thus, OSA affects a considerable proportion of the general population worldwide and is associated with an elevated public health burden, and empirical evidence leading to a better understanding of the burden of OSA is essential to guide prevention strategies. In Kuwait, obesity, a major risk factors for OSA, is highly prevalent and has been estimated to affect 40.3% (men: 36.5%; women: 44.0%) of adults (22). Hence, such an elevated prevalence of obesity may correlate with higher burden of OSA among adults in Kuwait. Given the scarcity of epidemiologic data on the OSA burden in Kuwait, this study sought to estimate the prevalence of those categorized as being at “high risk for OSA” using the Berlin Questionnaire among a working population in Kuwait and to ascertain the associated risk factors and comorbidities.

## Materials and Methods

### Study Setting, Design, and Participants

A population-based cross-sectional study was conducted to estimate the prevalence of being at a high-risk for OSA and to determine the potential associated risk factors and comorbidities among a working population in Kuwait. Study subjects were recruited from governmental/public workplaces (ministries/authorities) across Kuwait to represent working adults. From a comprehensive list of all the ministries and public authorities in Kuwait, seven venues were randomly selected using random digits. Subsequently, permissions were obtained to gain access to the selected venues, and employees (*n* = 651, aged 21–60 years) at the respective workplaces were enrolled as a convenience sample between December 2014 and January 2015. Hence, available and willing employees at the time of the study were enrolled. The study was approved by the Health Sciences Center Ethics Committee for Student Research at Kuwait University. Written informed consent was obtained from each study participant prior to enrolment. The study was conducted in accordance with the principles and guidelines of the Declaration of Helsinki for medical research involving human subjects.

### Study Questionnaires and Variable Definitions

Study participants were asked to complete the study questionnaire, which collected information on demographic characteristics, including height and weight, history of clinical conditions, lifestyle factors, and behavioral habits. In addition, the Berlin Questionnaire was used to ascertain each participant's OSA risk status (23), and the Insomnia Symptom Questionnaire (ISQ) was used to determine each subject's insomnia status (24). The study questionnaire (including the Berlin Questionnaire and the ISQ) was develop using English language and then translated into Arabic language. The comprehensibility and meaning of the translated questionnaire were pre-tested by 10 Arabic and English speaking subjects, and modifications were made as necessary. The final version of the Arabic questionnaire was back translated to English by an independent bilingual person to ensure neutral and valid translation. The back translated version was highly comparable to the original English language questionnaire. The used Arabic-translated Berlin Questionnaire is provided as an online supplementary material (see Supplementary Material).

Body mass index (BMI) was calculated as the weight in kilograms divided by the height in meters squared (kg/m^2^). Standard BMI groupings were applied, which included the following: underweight (BMI <18.5), normal weight (BMI 18.5–24.9), overweight (BMI 25.0–29.9), obesity class I (BMI 30.0–34.9), and obesity class II/III (BMI ≥35) (25). The underweight category was combined with the normal weight category, as only 15 (2.4%) subjects were classified as being underweight. Cigarette smoking was self-reported based on the following three categories: “never smoked,” “former smoker” (did not smoke cigarettes in the past 30 days), and “current smoker” (any cigarette smoking in the past 30 days). The frequency in which the subjects engaged in vigorous physical activities was assessed by the following question: “How many times a week do you engage in vigorous physical activity long enough to make you breathe hard?” The duration of television viewing was assessed by the following question: “During a normal week, how many hours a day (24 h) do you watch television?” Each participant's self-perceived physical health was assessed by asking the following question: “How would you rate your physical health?” The possible responses included “excellent,” “very good,” “good,” “fair,” and “poor.” The average number of daily hours of paid work was reported by each participant. Similarly, the average number of hours of nightly sleep during a regular working week was also reported.

With regard to existing health conditions, participants were asked the following question: “Has a doctor, nurse, or other health care provider ever told you that you have any of the following health conditions?” The listed conditions included diabetes, hypertension, hyperlipidemia, asthma, depression, and anxiety disorders. Moreover, the suffering of insomnia was ascertained based on participants' responses to the ISQ. Briefly, the ISQ is a 13-item self-report instrument designed to identify insomnia according to the following three sleep-related domains: (1) the presence of a complaint of difficulty initiating or maintaining sleep, or feeling that the sleep was nonrestorative or unrefreshing; (2) the frequency of sleep-related complaints and the duration of these symptoms; and (3) the severity of daytime impairment related to the sleep complaint(s) (24). If the participant met the criteria for all three domains, they were identified as having insomnia disorder; otherwise, they were not (24).

The Berlin Questionnaire, a widely applied instrument, was used to identify subjects who were at either a high risk or a low risk for developing OSA based on three symptom categories that inquire about known risk factors for OSA (23), namely snoring behavior, sleepiness or fatigue during waking hours, and the presence of obesity (i.e., BMI ≥ 30 kg/m^2^) or hypertension (self-reported). A “high risk for OSA” classification was assigned if the criteria for two or more categories were met, whereas a “low risk for OSA” classification was assigned if the criteria of either one or no categories was fulfilled (23).

### Statistical Analysis

Analyses were conducted using SAS 9.4 (SAS Institute, Cary, NC, USA). The statistical significance level was set at α = 0.05 for all association analyses. Descriptive analyses were conducted to calculate frequencies and proportions of categorical variables. Chi-square (χ^2^) tests were used to assess whether prevalence estimates of a “high risk for OSA” differed across categories of sex, age groups, BMI, educational attainment, and monthly household income. Moreover, linear trends in the prevalence of a “high risk for OSA” classification across categories of lifestyle and behavioral factors, perceived physical health, working hours, and sleep duration were assessed using the Cochran-Armitage test for trend.

Adjusted associations were assessed by applying a modified Poisson regression with robust variance estimation using the GENMOD procedure in SAS 9.4 to estimate and infer the adjusted prevalence ratios (aPRs) and their 95% confidence intervals (CIs) (26). We evaluated the associations between being at a “high risk for OSA” (outcome variable) and cigarette smoking status, frequency of vigorous physical activity, duration of weekly television viewing, self-perceived physical health, hours of daily work, and duration of nightly sleep (exposure variables). Moreover, we evaluated the associations between being at a “high risk for OSA” (exposure variable) and health conditions (outcome variables) while statistically adjusting for the effects of demographic characteristics, as well as lifestyle and behavioral factors.

## Results

### Description of the Study Sample

In total, 651 subjects (233 males and 418 females) were enrolled in the study, with a mean (standard deviation) age of 34.0 (9.1) years. Based on the BMI categories, 13.7% (85/620) and 8.1% (54/620) of the study participants were classified as belonging to obesity class I and obesity class II/III, respectively (Table 1). Moreover, most of the study participants reported having attained a university degree or higher (56.6%, 388/651; Table 1).

**Table 1:** Characteristics of study participants in the total study sample and according to the risk of obstructive sleep apnea (OSA).

**Variables**	**Total study sample (*n =* 651), % (*n*)**	**High risk for OSA**	
		**% (n/total)**	*P*-value^*^
**Sex**			
Male	35.8 (233)	24.0 (56/233)	0.053
Female	64.2 (418)	17.7 (74/418)	
**Age groups (years)**			
21–29	39.1 (253)	10.7 (27/253)	<0.001
30–39	37.8 (245)	17.6 (43/245)	
40–49	14.8 (96)	39.6 (38/96)	
≥50	8.3 (54)	40.7 (22/54)	
Missing, (*n*)	(3)	–	
**Body mass index groups (BMI) (kg/m**^**2**^**)**			
Underweight/normal weight^†^ (≤ 24.9)	43.7 (271)	6.6 (18/271)	<0.001
Overweight (25.0–29.9)	34.5 (214)	13.1 (28/214)	
Obesity class I (30.0–34.9)	13.7 (85)	57.7 (49/85)	
Obesity class II/III (≥35)	8.1 (50)	62.0 (31/50)	
Missing, (*n*)	(31)	–	
**Educational attainment**			
High school degree or less	9.2 (60)	38.3 (23/60)	<0.001
Diploma	31.2 (203)	18.7 (38/203)	
University degree or higher	59.6 (388)	17.8 (69/388)	
**Monthly household income (KWD)**			
<1,000	35.9 (234)	24.4 (57/234)	0.069
1,000–1,999	37.2 (242)	16.1 (39/242)	
2,000–2,999	14.5 (94)	23.4 (22/94)	
≥3,000	12.4 (81)	14.8 (12/81)	

*Kg, kilograms; m^2^, meter squared; KWD, Kuwaiti Dinar*.

* *Calculated using the chi-square test to compare proportions*.

† *The underweight group (BMI <18.5) was combined with the normal weight group (BMI between 18.5 and 24.9) because only 15 (2.4%) study participants were classified as being underweight*.

### Prevalence of the “High Risk for OSA” Classification

The prevalence of a “high risk for OSA” classification, as identified by the Berlin Questionnaire, was estimated to be 20.0% (130/651; 95% CI: 16.9–23.0%) in the total study sample. The prevalence of a “high risk for OSA” classification was higher in males (24.0%) than in females (17.7%, *P* = 0.053; Table 1), though this difference did not gain statistical significance. Moreover, the prevalence of a “high risk for OSA” classification increased with age, reaching as high as 40.7% among those aged ≥50 years (*P* < 0.001). Similarly, a “high risk for OSA” classification increased as BMI increased, with 62.0% of those classified as belonging to obesity class II/III being at “high risk for OSA” (*P* < 0.001; Table 1). With regard to educational attainment, the prevalence of a “high risk for OSA” classification was highest among subjects with a high school degree or less (38.3%, *P* < 0.001). However, the prevalence did not vary across income categories (*P* = 0.069; Table 1).

### Risk Factors for a “High Risk for OSA” Classification

Table 2 shows associations between different risk factors and being considered at a “high risk for OSA.” Compared with having never smoked, current smoking status was associated with a higher prevalence of being at a “high risk for OSA” (aPR = 1.58, 95% CI: 1.02–2.06). The frequency of vigorous physical activity was inversely associated with the prevalence of a “high risk for OSA” classification (P_trend_ = 0.001); however, after adjusting for possible confounding factors, the effect was not statistically significant (≥3 times per week vs. never/occasionally performing vigorous physical activity: aPR = 0.80, 95% CI: 0.48–1.33; Table 2). A longer daily duration of television viewing was associated with a higher prevalence of a “high risk for OSA” classification (≥5 h vs. <1 h per day: aPR = 1.76, 95% CI: 1.10–2.81). Moreover, lower self-perceived physical health was associated with increased prevalence of being at a “high risk for OSA” (fair/poor vs. excellent: aPR = 2.11, 95% CI: 1.15–3.87). As average daily working hours increased the prevalence of being at a “high risk for OSA” increased, with 17.5% of those who reported seven or fewer daily working hours being considered at a high risk compared to 40.0% of those who reported nine or more daily working hours (P_trend_ < 0.001). A longer average nightly sleep duration was associated with a reduced prevalence of a “high risk for OSA” classification (≥8 h vs. ≤6 h: aPR = 0.64, 95% CI: 0.40–0.98; Table 2).

**Table 2:** Associations of lifestyle (behavioral) factors, perceived physical health, working hours, and sleep duration with risk of obstructive sleep apnea (OSA).

	**Total Study sample, % (*n*/total)**	**High risk for OSA**	
		**% (n/total)**	aPR^*^ (95% CI)
**Cigarette smoking status**			
Never smoked	75.7 (493/651)	17.0 (84/493)	1.00 (Reference)
Former smoker	8.0 (52/651)	25.0 (13/52)	1.42 (0.90–2.78)
Current smoker	16.3 (106/651)	31.1 (33/106)	1.58 (1.02–2.06)^†^
${\text{P}}_{\text{trend}}^{§}$	–	<0.001	–
**Vigorous physical activity per week**			
Never/occasionally	44.1 (287/651)	25.8 (74/287)	1.00 (Reference)
Once or twice per week	38.9 (253/651)	16.2 (41/253)	0.75 (0.55–1.03)
Three or more times per week	17.0 (111/651)	13.5 (15/111)	0.80 (0.48–1.33)
${\text{P}}_{\text{trend}}^{§}$	–	0.001	–
**Daily hours of television viewing**			
Less than one hour	34.6 (225/650)	14.7 (33/225)	1.00 (Reference)
More than one but less than three hours	44.2 (287/650)	21.6 (62/287)	1.19 (0.84–1.66)
More than three but less than five hours	13.2 (86/650)	22.1 (19/86)	1.46 (0.92–2.32)
Five or more hours	8.0 (52/650)	30.8 (16/52)	1.76 (1.10–2.81)^‡^
${\text{P}}_{\text{trend}}^{§}$	–	0.006	–
**Self-perceived physical health**			
Excellent	23.9 (155/648)	11.0 (17/155)	1.00 (Reference)
Very good	42.1 (273/648)	13.2 (36/273)	1.06 (0.64–1.74)
Good	28.7 (186/648)	32.3 (60/186)	1.58 (0.97–2.56)
Fair/poor	5.3 (34/648)	50.0 (17/34)	2.11 (1.15–3.87)^‡^
${\text{P}}_{\text{trend}}^{§}$	–	<0.001	–
**Average daily hours of work**			
Seven or fewer hours	73.8 (479/649)	17.5 (84/479)	1.00 (Reference)
Eight hours	20.0 (130/649)	23.1 (30/130)	0.89 (0.63–1.24)
Nine or more hours	6.2 (40/649)	40.0 (16/40)	1.49 (0.96–2.29)
${\text{P}}_{\text{trend}}^{§}$	–	<0.001	–
**Average sleep duration in hours per night**			
Six or fewer hours	66.8 (434/650)	24.2 (105/434)	1.00 (Reference)
Seven hours	19.1 (124/650)	9.7 (12/124)	0.44 (0.27–0.72)^‡^
Eight or more hours	14.1 (92/650)	14.1 (13/92)	0.64 (0.40–0.98)^†^
${\text{P}}_{\text{trend}}^{§}$	–	0.002	–

*aPR, Adjusted prevalence ratio; CI, Confidence interval*.

* *Adjusted for sex, age, body mass index, educational attainment, monthly household income, and all variables shown in the table*.

† *P < 0.05*.

‡ *P < 0.01*.

§ *Calculated using the Cochran-Armitage test for a trend in proportions*.

### Health Conditions in Relation to a “High Risk for OSA” Classification

The associations between various health conditions and being considered at a “high risk for OSA” are shown in Table 3. Insomnia disorder was more prevalent among subjects who were classified as being at “high risk for OSA” compared to those classified as being at “low risk for OSA” (24.6% vs. 11.7%, aPR = 2.83, 95% CI: 1.81–4.41). Moreover, a “high risk for OSA” as compared to a “low risk for OSA” was associated with a higher prevalence of diabetes (aPR = 1.94, 95% CI: 1.15–3.27), hypertension (aPR = 3.00, 95% CI: 1.75–5.16), and depression (aPR = 4.47, 95% CI: 1.80–11.08). In contrast, there was no association between the risk for OSA and hyperlipidemia, asthma, or anxiety disorders (Table 3).

**Table 3:** Associations of obstructive sleep apnea (OSA) risk with comorbid health conditions.

**Health condition**	**Risk for OSA**		aPR^*^ (95% CI)	***P*-value**
	**Low risk, % (*n*/total)**	**High risk, % (*n*/total)**		
Insomnia	11.7 (61/521)	24.6 (32/130)	2.83 (1.81–4.41)	<0.001
Diabetes	6.4 (33/520)	16.2 (22/130)	1.94 (1.15–3.27)	0.012
Hypertension	5.8 (30/520)	33.9 (44/130)	3.00 (1.75–5.16)	<0.001
Hyperlipidemia	15.0 (78/520)	26.9 (35/130)	1.12 (0.76–1.67)	0.564
Asthma	12.7 (66/520)	13.9 (18/130)	1.49 (0.78–2.89)	0.229
Depression	4.0 (21/521)	7.7 (10/130)	4.47 (1.80–11.08)	0.001
Anxiety disorders	8.5 (44/520)	13.1 (17/130)	1.41 (0.70–2.83)	0.331

*aPR, Adjusted prevalence ratio; CI, Confidence interval*.

* *Adjusted for sex, age, body mass index, educational attainment, monthly household income, cigarette smoking status, vigorous physical activity status, daily hours of television viewing, self-perceived physical health, average daily hours of work, and average nightly sleep duration in hours*.

## Discussion

The current study estimated the prevalence of a “high risk for OSA” classification among a working population in Kuwait for the first time, determined the associated risk factors, and identified several coexisting health conditions. This investigation showed that 20.0% of the enrolled subjects were at high risk for OSA, as measured by the Berlin Questionnaire. A high risk for OSA was more prevalent among males, older participants, obese subjects, and those with low educational attainment. Moreover, current smoking, never/occasionally being involved in vigorous physical activity, longer daily hours spent watching television, a lower level of self-perceived physical health, increased hours of daily work, and a short nightly sleep duration were associated with an increased prevalence of being classified at a high risk for OSA. Certain health conditions, including insomnia disorder, diabetes, hypertension, hyperlipidemia, and depression were more prevalent among participants classified as being at high risk for OSA compared to those at low risk for OSA.

A prior systematic review of the literature that included studies that objectively measured OSA among adults in the general population using laboratory instruments showed wide variations in the prevalence of OSA, with estimates of mild OSA (AHI ≥ 5 to <15) ranging from 9 to 38%, and estimates of moderate-to-severe OSA (AHI ≥15) varying from 6 to 17% (9). Another systematic review focusing on studies published among Asian adults reported that OSA prevalence ranged from 3.7 to 97.3% (27). At the regional level, a study based on a large sample of Saudi school employees estimated the prevalence of OSA to be 8.8% (12.8% in men and 5.1% in women) based on polysomnography assessments (28). Such a wide variability in the reported prevalence of OSA can be partially explained by the method of ascertainment (objective [laboratory-based] vs. subjective [questionnaire-based]) and the study population's risk for OSA (i.e., pretest probability of OSA). In the current study, 20.0% of the participants were identified as being at high risk for OSA according to the Berlin Questionnaire criteria. This estimate is within the mild OSA prevalence range (9% to 38%) reported by Senaratna et al. (9). Moreover, our estimate is consistent with previously reported OSA prevalence estimates in general adult populations from studies that used the Berlin Questionnaire to ascertain OSA risk status. For example, the results of the National Sleep Foundation *Sleep in America 2005* poll showed that 26% of the study participants met the Berlin Questionnaire criteria for a high risk for OSA (29). Moreover, a nationwide survey conducted among the Korean adult population reported that 15.8% of the participants were classified as being at high risk for OSA based on the same Berlin Questionnaire definition (30). In a primary health care setting, a study conducted in the United Arab Emirates estimated the prevalence of a high risk for OSA to be 20.9% using the Berlin Questionnaire criteria (31). Nevertheless, comparing OSA prevalence estimates between populations should be interpreted with caution, as characteristics of study subjects and ascertainment methods could contribute to the observed inter-population heterogeneity.

In this report, a higher prevalence of a high risk for OSA was observed among male (sex-based difference did not gain statistical significance), older aged, and obese participants, which is in agreement with the existing literature (4–6, 32). Moreover, current smoking status compared to never having smoked was associated with an increased prevalence of being classified at a high risk for OSA among our study participants. This observation has been reported previously and is hypothesized to be explained by the potential health-related changes caused by smoking, including increases in sleep instability and airway inflammation (6, 15, 33). With regard to protective factors, we observed a lower prevalence of being classified at a high risk for OSA among participants who reported regular engagement in vigorous physical activity compared to those who were never/occasionally involved in such activities. This observation further highlights the positive effects of physical activity on general health and OSA risk. An analysis based on the Ontario Health Study reported that increased levels of vigorous-intensity activity and walking were associated with reduced OSA risk (34), which further corroborates our observation. In contrast, an increased number of daily hours spent watching television was associated with a higher prevalence of a “high risk for OSA” in the current report, even after adjusting for potential confounding factors, including obesity status. Increased television viewing is probably a surrogate marker of a sedentary lifestyle and unhealthy dietary intake (35, 36). Interestingly, we observed a positive association between the average number of daily hours spent working and a high risk for OSA, where those reporting at least nine daily hours of work had the highest prevalence of OSA risk. Such an observation could be explained by the potentially fewer hours spent sleeping among those performing longer work hours; however, the observed association was independent of the average number of nightly sleep hours reported by the participants. Hence, the factors underlying this observation warrant further investigation. Moreover, we reported that the prevalence of being at a high risk for OSA decreased as the average nightly sleep duration increased. In line with our observation, a prior study showed that the average sleep duration of the group at high risk for OSA (7.0 ± 1.4 h) was shorter than that of subjects in the group at low risk for OSA (7.4 ± 1.2 h, *P* < 0.001) (30). These observations further highlight the fact that reduced sleep duration is a characteristic of OSA that can lead to adverse health outcomes. A study from Muscat, Oman reported a high prevalence of relatively short night sleep duration (<7 h), which authors have indicated that such a nocturnal sleep duration is culturally influenced and is related to sleep patterns and behaviors that have been adapted recently by people in the region (37).

The current analysis also showed that being classified at a high risk for OSA was associated with several comorbid conditions. For instance, insomnia disorder was 2.83-times more prevalent among subjects at high risk for OSA compared to those at low risk for OSA. This observation is in agreement with prior investigations that indicated that the co-occurrence of insomnia disorder and OSA is common based on a large global meta-analysis (38). Also, we observed that subjects who were at high risk for OSA, compared to those at low risk for OSA, had a higher prevalence of diabetes (16.2 vs. 6.4%). This finding is also consistent with results of a meta-analysis of cohort studies that estimated the pooled relative risk for the association between OSA risk and diabetes development to be 1.40 (95% CI: 1.32–1.48) (39). The same meta-analysis reported a dose-effect relationship between the AHI value and diabetes risk (39). We also observed an association between OSA risk and hypertension, which is in agreement with prior investigations that have shown an increased risk of hypertension and cardiovascular disease among subjects with OSA (40, 41). In regard to psychological disorders, a higher prevalence of depression was reported by subjects classified as being at high risk for OSA compared to those at low risk for OSA. Although this observation has been reported previously, the directionality of this association remains unclear (20, 42). In general, the co-occurrence of health conditions with OSA can be partially explained by certain shared risk factors and pathophysiology; nevertheless, more investigations are needed to better understand the underlying mechanisms.

The strength of the current study was the enrollment of working subjects, which could more closely reflect the OSA burden in the general population than a clinical-based study sample. Moreover, recruiting participants from different places of work increased the representativeness of our sample. Nevertheless, non-probability-based sampling (i.e., convenience sampling) may limit the generalizability of our results. For instance, the proportion of female participants was higher than the proportion of male participants in our study sample (64.2 vs. 35.8%). Such a sex-related self-selection bias may reduce the external validity of our findings, but not the internal validity of the study. A further limitation was the questionnaire-based ascertainment of the participants' OSA risk status. We did, however, use the Berlin Questionnaire to do so, which a meta-analysis has shown to be an acceptable instrument for OSA ascertainment, with a pooled sensitivity of 0.76 (95% CI: 0.71–0.81) and pooled specificity of 0.59 (0.48–0.66) as compared to a laboratory-based assessment of OSA (13). Such results demonstrate an acceptable accuracy; however, misclassification of OSA risk in large epidemiologic studies using questionnaire-based assessments is inevitable; however, our estimated prevalence of being at a high risk for OSA is close to reported estimates from other countries, as were the associations we reported. This is an indication that the misclassification of OSA risk status, if any, was not substantial and did not influence the study's results. Moreover, selection bias cannot be excluded in cross-sectional studies; more specifically, self-selection bias, which can occur when more susceptible individuals participate in a study. It is also essential to note that our analysis aimed to assess concurrent associations between OSA risk status and different risk factors and health conditions, not to infer causal or temporal relationships.

In conclusion, this study is the first to estimate the prevalence of OSA risk among a working population in Kuwait and to assess its risk factors and comorbid conditions among this population. The findings of our study indicated that 1/5 of working adults are at high risk for OSA, which depends on an individual's personal characteristics and lifestyle factors. For instance, modifiable risk factors associated with elevated OSA prevalence included smoking, sedentary lifestyle (no physical activity and increased television viewing), increased working hours, and short nightly sleep duration. In addition, we demonstrated that being at high risk for OSA was associated with multiple coexisting health conditions, such as insomnia disorder, diabetes, hypertension, and depression. Overall, the results of this study demonstrated that OSA is common among a sample of working adults in Kuwait and is associated with elevated morbidity. To guide public health prevention efforts, future studies are needed to corroborate our findings and to assess the burden of OSA in different settings.

## Data Availability Statement

The raw data supporting the conclusions of this article will be made available by the authors, without undue reservation.

## Ethics Statement

The studies involving human participants were reviewed and approved by The Health Sciences Center Ethics Committee for Student Research at Kuwait University. The patients/participants provided their written informed consent to participate in this study.

## Author Contributions

HA-Q and HA-O conceptualized and designed the study, designed the data collection instrument, collected data, analyzed and interpreted the data, and drafted the manuscript. KA-H, FA-M, MA-M, and MA-A conceptualized and designed the study, designed the data collection instrument, collected data, contributed to data analysis and interpretation, and critically reviewed and revised the manuscript for important intellectual content. AM and AA contributed to conceptualization and design of the study, contributed to data analysis and interpretation, and critically reviewed and revised the manuscript for important intellectual content. AZ contributed to conceptualization and design of the study, contributed to designing the data collection instrument, supervised data collection, contributed to data analysis and interpretation, and critically reviewed and revised the manuscript for important intellectual content. All authors have reviewed, revised, and approved the final manuscript.

## Conflict of Interest

The authors declare that the research was conducted in the absence of any commercial or financial relationships that could be construed as a potential conflict of interest.
