# Supplementary material for: Prevalence, Risk Factors, and Comorbidities of Obstructive Sleep Apnea Risk Among a Working Population in Kuwait: A Cross-Sectional Study
Source: Front Neurol. 2021 Apr 6;12:620799. doi: 10.3389/fneur.2021.620799 (PMC8056027; doi:10.3389/fneur.2021.620799)
Supplement: Supplementary file 1 [file Data_Sheet_1.PDF]

Arabic Translation of the Berlin Questionnaire

استبيان برلين للاختناق التنفسي أثناء النوم

.1 العمر:______________سنة

.2 الجنس:

☐[1] ذكر
☐[2] انثى

.3 الطول: ________سم **الوزن:** _________كج

.4 هل تم اخبارك من قبل دكتور، ممرض، او اي عامل اخر بالقطاع الصحي بأنك مصاب بارتفاع ضغط الدم

☐[1] نعم ☐[0] لا

الأسئلة التالية متعلقة بأنماط النوم

.5 هل تشخر اثناء النوم؟

☐[1] نعم ☐[0] لا ☐[2] لا اعلم

اذا كنت تشخر، هل اخبرك شخصا ما:

.6 بأن شخيرك:

☐[1] اعلى بقليل من التنفس
☐[2] عال بدرجة مساوية للكلام
☐[3] اعلى من الكلام
☐[4] عال جدا، يمكن سماعه بالغرف المجاورة

.7 عدد المرات التي تشخر بها؟

☐[1] كل يوم تقريبا
☐[2] 3-4 مرات اسبوعيا
☐[3] 1-2 مرة اسبوعيا
☐[4] 1-2 مرة بالشهر
☐[5] لا اشخر

8. هل سبق ان ضايق شخيرك أشخاص اخرين؟

☐[1] نعم ☐[0] لا ☐[2] لا اعلم

9. هل لاحظ احدا بأنك تتوقف عن التنفس اثناء النوم؟

☐[1] كل يوم تقريبا
☐[2] 3-4 مرات اسبوعيا
☐[3] 1-2 مرة اسبوعيا
☐[4] 1-2 مرة بالشهر
☐[5] لم يلاحظ احد توقفي عن التنفس اثناء النوم

10. عدد المرات التي تشعر فيها بالتعب او الارهاق بعد استيقاظك من النوم؟

☐[1] كل يوم تقريبا
☐[2] 3-4 مرات اسبوعيا
☐[3] 1-2 مرة اسبوعيا
☐[4] 1-2 مرة بالشهر
☐[5] ولا يوم

11. اثناء يقظتك، هل تشعر بالتعب، الارهاق، او بأنك لست بأحسن حالاتك؟

☐[1] كل يوم تقريبا
☐[2] 3-4 مرات اسبوعيا
☐[3] 1-2 مرة اسبوعيا
☐[4] 1-2 مرة بالشهر
☐[5] ولا يوم

12. هل سبق ان غفيت او نمت اثناء قيادتك للمركبة؟

☐[1] نعم
☐[0] لا

<u>اذا اجبت ب"نعم" اذهب الى سؤال 13</u>

13. ما عدد المرات التي حصل فيها ذالك؟

☐[1] كل يوم تقريبا
☐[2] 3-4 مرات اسبوعيا
☐[3] 1-2 مرة اسبوعيا
☐[4] 1-2 مرة بالشهر
☐[5] ولا مرة
